# Recent Advances on Chitosan-Based Thermosensitive Hydrogels for Skin Wound Treatment

**DOI:** 10.3390/biology14060619

**Published:** 2025-05-27

**Authors:** Jin Wang, Lianghui Huang, Enguang Wu, Xiao Li, Yi Rao, Caiqing Zhu

**Affiliations:** 1 Nanchang Key Laboratory for Quality Evaluation of Medical Devices, Jiangxi Medical Device Testing Center, Nanchang 330001, China; 1020220803@jxstnu.edu.cn; 2 National Pharmaceutical Engineering Center for Solid Preparation in Chinese Herbal Medicine, Jiangxi University of Chinese Medicine, Nanchang 330006, China; 3 School of Pharmacy, Jiangxi Science and Technology Normal University, Nanchang 330013, China; hlh3986181@163.com (L.H.); wuenguang2018@163.com (E.W.); 18702619348@163.com (X.L.)

**Keywords:** chitosan, thermosensitive hydrogel, healing mechanism, wound dressing

## Abstract

Thermosensitive hydrogel, as a smart polymer material, showed great potential for application in the field of wound repair due to its unique external temperature responsiveness and excellent biocompatibility. Chitosan, a natural macromolecular polysaccharide derived from the deacetylation of chitin, possessed not only strong interactions with biomolecules such as DNA, proteins, and lipids, but also unique biocompatibility and degradability. Chitosan-based thermosensitive hydrogels, prepared by compounding chitosan with surfactants, underwent sol–gel phase transitions at varying external temperatures, which provided an ideal healing environment for wounds. This comprehensive review was initiated by elucidating the sol–gel phase transformation mechanism underlying thermosensitive hydrogels and the intricate process of wound repair. In addition, this review provided a detailed overview of the prevalent types of chitosan-based thermosensitive hydrogels, highlighting their unique characteristics and applications in different types of wound repair. Finally, the challenges and development directions of chitosan-based thermosensitive hydrogels in wound repair were discussed, aiming to provide theoretical support and practical guidance for their future applications in wound healing.

## Simple Summary

The motivation for this review stems from the gradually deepening understanding of the application potential of chitosan-based thermosensitive hydrogels in the field of wound repair and the mechanisms underlying wound healing. With the advancement of tissue engineering and regenerative medicine, modulating the wound microenvironment to promote healing has emerged as a pivotal objective. Chitosan-based thermosensitive hydrogels have garnered considerable attention due to their unique physicochemical properties, such as temperature-responsive gelation, biocompatibility, and tunable degradability. By systematically analyzing the phase-transition mechanism of thermosensitive hydrogels and the wound-healing process, this review sorts out the current types of chitosan-based thermosensitive hydrogels, emphasizing their characteristics and applications in different wound types. Additionally, it discusses the challenges and future development directions of chitosan-based thermosensitive hydrogels in wound repair. This work holds significant clinical value, as it not only deepens the understanding of chitosan-based thermosensitive hydrogels but also provides a theoretical foundation and technical pathways for the development of next-generation smart wound-repair materials.

## 1. Introduction

Wounds caused by infection, burns, or diabetes are common in clinical practice, and these wounds are usually difficult or even unable to heal due to excessive inflammation, oxidative stress, peripheral neuropathy, and impaired blood vessel formation [1,2]. It seriously affects the quality of life and physical and mental health of patients, and, at the same time, it also constitutes a heavy economic burden to society. Wounds are frequently accompanied by painful bleeding and extended healing times, causing prolonged suffering for patients. The application of suitable wound dressings during this period is able to expedite the healing process of skin wounds [3]. The wound-healing process is intricate and dynamic, encompassing different stages including hemostasis, inflammation, and proliferation, as well as tissue remodeling [4,5]. Hydrogels are hydrophilic polymer materials characterized by a three-dimensional network structure, which are synthesized through either the physical or chemical crosslinking of hydrophilic polymers [6]. They demonstrate exceptional biocompatibility and mechanical properties, as well as tissue adhesion through functional modifications, making them indispensable in the field of wound dressings [7,8]. In particular, hydrogel-based dressings have been demonstrated to expedite the wound-healing process by providing an optimal moist healing environment. A moist environment aids in maintaining proper hydration, promoting angiogenesis and collagen synthesis, and facilitating the debridement of necrotic tissue, thereby accelerating the overall healing process [7]. However, traditional hydrogel materials struggle to accurately integrate with surrounding wound tissue defects during medical clinical applications, and invasive implantation might lead to secondary wound damage [9,10]. Injectable hydrogels have emerged as a potential solution to these problems due to their advantages such as ease of operation, minimally invasive administration, and low injection costs [11,12,13]. They fill small and irregular wounds, are implanted into difficult-to-reach treatment sites, and gradually undergo sol–gel phase transitions under physiological conditions for precise wound treatment [14,15].

While previous studies have explored injectable hydrogels, this review distinguishes itself by focusing specifically on thermosensitive hydrogels and their application in wound healing. Injectable hydrogels are able to respond to various environmental stimuli such as pH, temperature, electric field, pressure, and ionic strength for sol–gel phase transitions [16,17,18]. Among these, temperature is relatively easy to access and control. Moreover, pathological changes in biological tissues are often accompanied by body temperature variations [19]. Thermosensitive hydrogels, initially in a liquid (sol) state, are able to achieve a reversible sol–gel state transition through the subtle control of environmental temperature after injection into the body [20]. Thermoresponsive hydrogels are categorized into two types based on their expansion mechanisms: thermally expanding hydrogels that expand upon heating and thermally contracting hydrogels that contract upon heating [21]. Thermally expanding hydrogels are in a contracted state when the temperature is below the phase transformation temperature, but they expand when the temperature exceeds this threshold. Conversely, thermally contracting hydrogels are in an expanded state below the phase-transition temperature and contract as the temperature rises above it. Compared to traditional hydrogels, thermosensitive hydrogels offer significant advantages in the field of wound repair, as they are able to undergo a transition from the sol to gel state in response to temperature changes [22].

**Figure 1:**
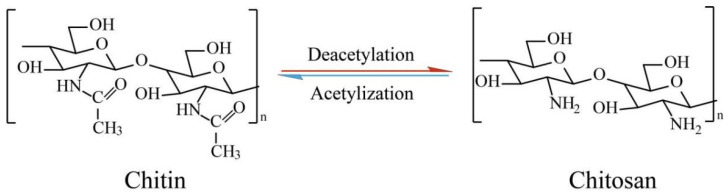
The structure of chitin and chitosan.

Thermoresponsive hydrogels are primarily derived from natural polymers and synthetic polymers [23]. Compared to synthetic polymer hydrogels, natural polymer hydrogels have gained favor among researchers due to their advantages in resources, structure, and performance [24]. Chitosan is an alkaline amino polysaccharide prepared by the deacetylation of chitin (Figure 1). Its structure is composed of an *N*-acetylglucosamine fragment linked by a β-1,4 glycosidic bond [25]. Compared with other natural polymers, chitosan is the only cationic polysaccharide and antibacterial activity in nature, and thus has been widely used in wound healing [23]. However, due to its relatively slow thermal response and low mechanical strength, chitosan often requires modification, typically by combination with other materials, to enhance its properties. Common examples of chitosan-based thermosensitive hydrogels include chitosan–sodium glycerophosphate hydrogel, hydroxybutyl chitosan hydrogel, chitosan/polyol-polymer hydrogel, chitosan/amphiphilic polymer hydrogel, and chitosan/alkaline inorganic salt hydrogel [26].

Previous studies on chitosan-based thermosensitive hydrogels have reported on their basic properties and some applications. However, this review offers a more comprehensive and in-depth analysis. This review first outlined the sol to gel phase-transformation mechanism of thermosensitive hydrogels and illustrated their response mechanism to changes in external temperature. Subsequently, a comprehensive review of the natural process of wound repair was provided, revealing the key steps and influencing factors in wound healing. In addition, the review focused on reviewing the currently commonly used types of chitosan-based thermosensitive hydrogels and explored their applications in different types of wound healing. Finally, the challenges faced by chitosan-based thermosensitive hydrogels in the field of wound healing, such as the complexity of the preparation process, further improvement of performance, and safety evaluation of clinical applications, were discussed. Corresponding development directions were proposed to provide theoretical support and practical guidance for the future application of chitosan-based thermosensitive hydrogels in the field of wound healing.

## 2. The Sol–Gel Phase-Transformation Mechanism of Thermosensitive Injectable Hydrogels

**Figure 2:**
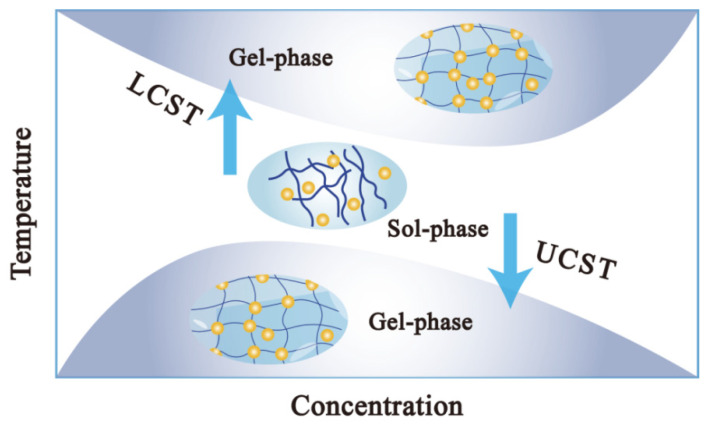
Schematic diagram of LCST-type phase transformation and UCST-type phase transformation.

The fundamental aspect of the sol–gel phase-transformation mechanism in thermosensitive hydrogels resides in the change in hydrophilic and hydrophobic balance within polymer molecular chains [27,28]. Amphiphilic thermosensitive polymers, consisting of both hydrophobic and hydrophilic segments, undergo self-assembly into micelles in aqueous environments, driven by the hydrophobic effect exhibited by their hydrophobic segments [29]. As the temperature increases, the hydrogen bonding between the surface of the polymer micelles and the water molecules is weakened, which results in the thinning of the hydration layer around the micelles, and the aggregation of the micelles with each other leading to the formation of macroscopic hydrogels [30]. As shown in Figure 2, in the process of temperature changes from low to high, the polymer transitions from a fully dissolved state to a partially dissolved state, and its state also changes from a sol state (sol phase) to a gel state (gel phase) [31]. This polymer sol–gel phase transformation is called a lower critical solution temperature (LCST)-type phase transformation (commonly represented by LCST for the phase-transition temperature or Tsol–gel for the gelation temperature). However, a small number of thermosensitive hydrogels exhibited the opposite behavior with temperature changes, known as an upper critical solution temperature (UCST)-type phase transformation (commonly represented by UCST for the phase-transformation temperature or Tgel-sol for the sol temperature) [32]. Polysaccharides such as carrageenan, agarose, and gellan gum, as well as their derivatives, typically underwent UCST-type phase transitions and were commonly adapted as emulsifiers as well as thickeners in the food industries [33]. UCST-type thermosensitive hydrogels require higher temperatures to maintain their solution state, but excessively high temperatures result in a reduction in the viability of cells and biological tissues, while LCST-type thermosensitive hydrogels are free-flowing solutions at room temperature, which are more suitable for injectable hydrogel systems, and transform into gels at physiological temperatures (36–37 °C) [34]. Therefore, LCST-type hydrogels are garnering increased attention in the realm of biomedical applications. This review focused on discussing the sol–gel phase-transition mechanism of LCST-type hydrogels. The sol–gel phase-transformation mechanism of LCST-type injectable hydrogels could be illustrated from both molecular as well as thermodynamic perspectives [35].

### 2.1. Molecular Level

At the molecular level, hydrophilic interactions take precedence when the ambient temperature falls below the LCST of the polymer. These interactions cause the hydrophilic groups on the polymer’s molecular chains to establish hydrogen bonds with water molecules, ultimately resulting in a sol state. Once injected into an animal’s body, as the temperature surpasses the LCST, hydrophobic interactions emerge as the dominant force. Consequently, the polymer’s molecular chains engage in interactions and self-assemble via their hydrophobic segments, forming larger aggregates and undergoing a gelation phase transition [36]. Typically, the morphology of the hydrogel undergoes reversible transitions between the sol state and the gel state due to the dynamic interactions between the polymer molecular chains as well as water molecules. Therefore, the fundamental impetus behind the sol–gel phase transformation in thermosensitive polymers is a change in interactions among the polymer’s hydrophobic segments, which is induced by temperature variations [37].

### 2.2. Thermodynamic Angle

Thermodynamically, according to the Gibbs free energy formula (ΔG = ΔH − TΔS), the associative free energy of polymer molecular chains is closely related to enthalpy, entropy, and temperature [38]. In the amphiphilic thermosensitive polymer–water system, when T < LCST, the polymer molecular chain unfolds and generates hydrogen bonds with water molecules, contributing to ΔH > 0. Meanwhile, the orderly arrangement of water molecules around the polymer molecular chain leads to ΔS < 0, further causing ΔG > 0, and the polymer dissolves in water in a sol state. When T > LCST, the entropy of the system aggrandizes as well as dominates, leading to ΔH < TΔS, ultimately resulting in ΔG < 0, which facilitates the association of polymer molecular chain and presents a gel state. It is seen that the sol–gel phase transformation of thermosensitive polymers originates from the change in entropy caused by temperature changes [39,40].

## 3. Wound-Repair Process

**Figure 3:**
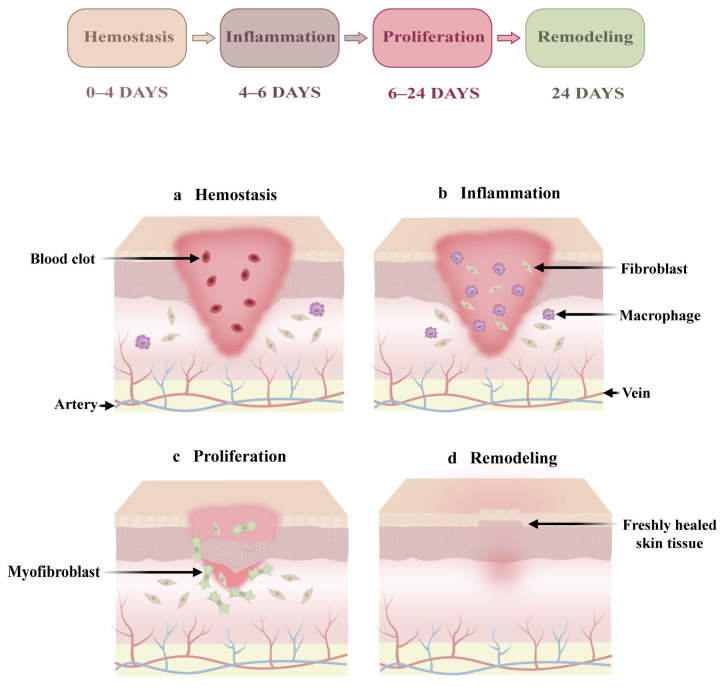
Wound-repair process.

Skin wounds are a common occurrence caused by surgical operation, empyrosis, and chronic ulcers, as well as traumatic injuries [21]. However, the wound-repair process is a complex physiological phenomenon influenced by many factors [41]. Typically, the complete wound-repair process consists of four stages: hemostasis, inflammation, proliferation, and tissue remodeling (Figure 3).

### 3.1. Hemostasis

Skin wounds cause bleeding, and hemostasis begins the repair process. When skin bleeds, the body’s spontaneous hemostatic mechanisms activate [42]. Vasoconstriction and platelet aggregation happen first, then the coagulation system is activated, converting fibrinogen to fibrin to form a clot and stop bleeding [43]. For large wounds, the body’s natural mechanisms often cannot stop the bleeding. Uncontrolled bleeding leads to complications like infection, hypothermia, hypotension, and shock, hindering healing and raising morbidity and mortality if not treated promptly. Traditional hemostatic materials like bandages and gauze, which use direct pressure, are easy to make, cost effective, and reusable [44]. But they are prone to infection when in contact with blood or tissue fluid, can tear, causing discomfort and prolonging healing, and may not fit irregular, deep, or narrow wounds well [45]. Besides compression, other hemostatic techniques like local devices, adhesives, and sealants are effective in surgery and emergencies [46]. Hemostatic agents enhance clotting, and adhesives bind tissues and vessels [47]. However, fibrin-based sealants have poor adhesion and are affected by blood perfusion, failing to stop bleeding and increasing infection risk. Strong adhesives like cyanoacrylates cause allergies, heat during solidification, and have toxic degradation products [48]. So, there is an urgent need for safe, fast, and efficient hemostatic materials.

### 3.2. Inflammation

**Figure 4:**
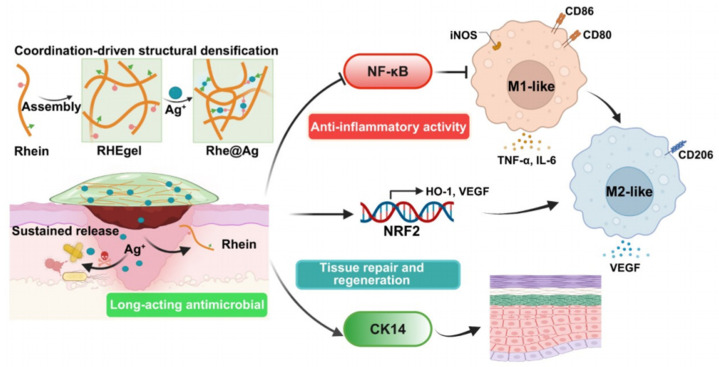
Illustration of Rhe@Ag hydrogel for synergistic antibacterial and anti-inflammatory therapy in wound healing [51].

The inflammatory phase constitutes the second stage of skin repair. It is a vital immune response that helps the body survive infections as well as tissue damage and maintain normal tissue homeostasis. In this phase, inflammatory cells remove bacteria and necrotic tissues [49]. However, it is important to note that excessive and prolonged inflammation are detrimental rather than beneficial [50]. To repair damaged skin, a wound dressing with excellent anti-inflammatory effects is needed. For example, Liang et al. developed a novel Rhe@Ag hydrogel, which was constructed through the self-assembly of the natural small molecule drug Rhein and the incorporation of silver ions (Ag^+^). Rhein promoted skin regeneration and accelerates wound healing by reprogramming M1 to M2 macrophages. Mechanistically, Rhein exerted anti-inflammatory effects via NRF2/HO-1 activation and NF-κB inhibition. Therefore, Rhe@Ag hydrogel synergistically combined Ag^+^’s antibacterial properties with Rhein’s anti-inflammatory and regenerative functions, providing a new strategy for wound management with dual roles (Figure 4) [51].

### 3.3. Proliferation

**Figure 5:**
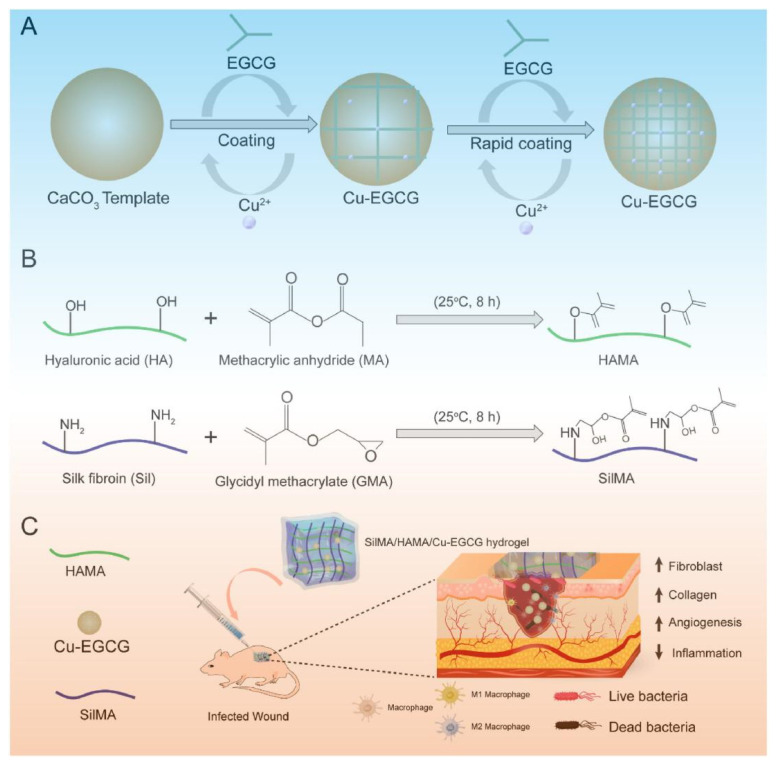
(**A**) A schematic of the synthesis of Cu-EGCG. (**B**) A schematic illustration showing the synthesis of HAMA and SilMA. (**C**) The application of the SilMA/HAMA/Cu-EGCG hydrogel for infected wound healing and skin reconstruction [58].

The proliferation phase is the third stage of skin repair, marked by tissue regeneration and granulation tissue formation [52]. Epithelial cells proliferate and move to the wound site, while inflammatory cells, fibroblasts, and new capillaries work together to form granulation tissue. Studies show that hydrogels boost skin wound repair by stimulating the proliferation of inflammatory cells, fibroblasts, and capillaries [53,54,55,56,57]. For instance, Liu et al. constructed Cu-Epigallocatechin-3-gallate (Cu-EGCG) nano-capsules and a hydrogel with a dual-network structure formed by cross-linking hyaluronic acid methacrylate (HAMA) with methacrylose-modified silk fibroin (SilMA). Cu-EGCG nano-capsules was loaded into SilMA/HAMA hydrogel wound dressings to form HAMA/SilMA/Cu-EGCG hydrogel, which continuously release EGCG and copper ions, promoted the proliferation of fibroblasts and collagen deposition, accelerated dre-epithelialization and neovascularization, and significantly promoted the healing of full-thickness skin wounds (Figure 5) [58].

### 3.4. Remodeling

**Figure 6:**
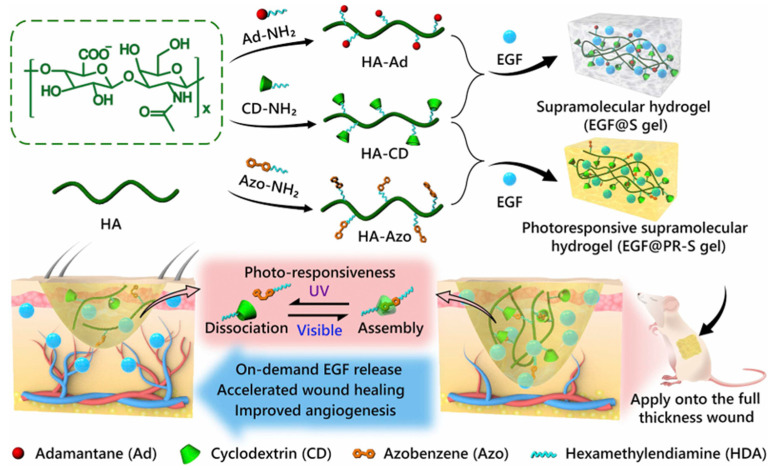
A schematic illustration of the synthetic route of supramolecular hydrogels and their application as controlled delivery systems for accelerated wound healing [62].

In the final remodeling phase, platelets in the blood clot release growth factors like platelet-derived growth factor (PDGF) as well as transforming growth factors (TGF-α and TGF-β) to aid wound healing [59]. PDGF promotes angiogenesis by attracting fibroblasts and stimulating collagen deposition, vital for connective tissue repair. This leads to the regeneration of new epidermal and dermal layers, completing skin repair [60]. Growth factors in skin wounds are vital to accelerating repairing, particularly by adjusting the proliferation, epithelialization, and remodeling of the extracellular matrix, and the angiogenesis of keratinocytes as well as fibroblasts [61]. For instance, Zhao et al. made photo-responsive supramolecular polysaccharide hydrogels via host–guest interactions between azobenzene and β-cyclodextrin groups on hyaluronic acid chains. Using azobenzene’s photoisomerization under different wavelengths, a hydrogel with a dynamic spatial network crosslink density was created. Under ultraviolet (UV) light, the loosened hydrogel quickly released EGF, reshaping the extracellular matrix and speeding up wound healing (Figure 6) [62].

## 4. Chitosan-Based Thermosensitive Hydrogels

### 4.1. Chitosan–Sodium Glycerophosphate Thermosensitive Gel

**Figure 7:**
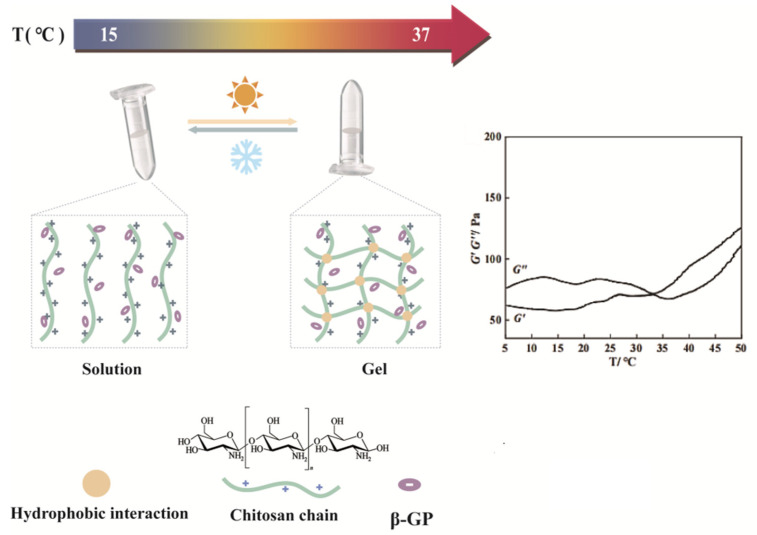
Formation process of chitosan and β-glycerophosphate hydrogels.

The mixture of chitosan and sodium glycerophosphate showed remarkable thermosensitive properties [63]. This solution undergoes a temperature-responsive phase transition. Specifically, it remains stable in a liquid state at room temperature over an extended period but undergoes a rapid transformation into a gel state upon reaching body temperature (37 °C). Polyols influence chitosan, causing this phase shift. They create a protective and water-repellent barrier around chitosan chains through weak intermolecular forces such as hydrogen bonds. When the temperature rises, the polyol layer is progressively stripped away, enabling the polymers to achieve an equilibrium state via stronger hydrophobic interactions and leading to gelation [64]. The molecular mechanism behind this gelation involves complex interactions between chitosan, β-glycerophosphate, and water. β-glycerophosphate plays a crucial role as an electrostatic repellant, enhancing hydrogen-bond connections between chitosan chains [65]. Meanwhile, the electrostatic attraction between the ammonium groups of chitosan and the phosphate groups of β-glycerophosphate, as well as the hydrophobic interactions between chitosan molecules, collectively promote the formation of the gel (Figure 7).

The stability and viscosity properties of chitosan–sodium glycerophosphate solutions are closely related to the degree of deacetylation of chitosan [66]. Specifically, a lower degree of deacetylation of chitosan results in a solution that is able to maintain a stable liquid state under temperature changes for a longer period, and its viscosity remains constant for a longer time. This was further confirmed by Chenite et al. who prepared a pH-neutral chitosan–sodium glycerophosphate complex by neutralizing chitosan using sodium glycerophosphate and found that the rate of gel formation in the solution was mainly dependent on the degree of deacetylation of chitosan, and that chitosan with a high degree of deacetylation was more likely to be attached to the amino group of sodium glycerophosphate, leading to accelerated gel formation [67]. Additionally, Deng et al. revealed that the ratio of chitosan to β-glycerophosphate sodium affected the gelation temperature, pore size, and degradation rate, as well as that the gelation temperature decreased with an increase in the content of β-glycerophosphate [68].

### 4.2. Hydroxybutyl Chitosan Hydrogel

**Figure 8:**
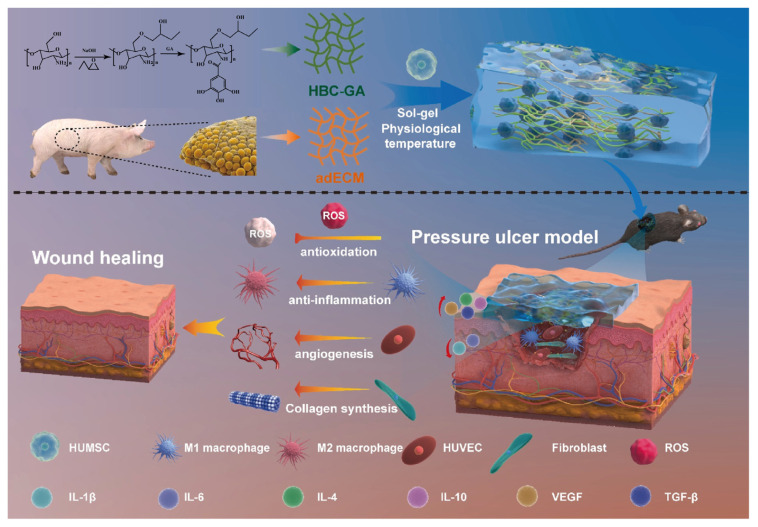
The mechanism of adEHG hydrogel in promoting full-thickness pressure ulcer healing [73].

Hydroxybutyl chitosan (HBC), one of the most important chitosan ethers, is synthesized by coupling hydroxybutyl with hydroxyl and amino groups of the chitosan skeleton through etherification reaction, which endows it with richer and more diverse functional properties [69]. HBC exhibits unique thermosensitive properties in aqueous solutions. It has a low critical solution temperature of approximately 19 °C, which allows it to rapidly form stable gels within a very short time span under changes in ambient temperature [70]. This temperature sensitivity not only grants HBC tremendous potential in drug delivery and biomaterial preparation, but also opens up new avenues for its application in the biomedical field. Based on the excellent performance of HBC, a series of novel biomaterials have been developed. These materials have been widely used in various applications such as preventing post-surgical adhesions, wound dressings, arterial embolization agents, tissue engineering scaffolds, delivery vehicles, and cell therapy agents [71,72]. Tang et al. developed a thermosensitive injectable hydrogel (adEHG) that combined gallic acid-modified hydroxybutyl chitosan (HBC-GA) with soluble extracellular matrix (adECM). adEHG hydrogel had excellent physical and chemical properties, which protected stem cells from oxidative stress and enhanced their therapeutic effect by eliminating ROS. In addition, adEHG hydrogel promoted angiogenesis, cell proliferation, and collagen deposition, and further enhanced inflammatory regulation and wound healing by continuously releasing therapeutic factors and cells (Figure 8) [73].

### 4.3. Chitosan/Polysol-Polymer Hydrogel

Polysol polymers are characterized by a number of hydroxyl groups in their main chains, such as polyethylene glycol (PEG) and polyvinyl alcohol (PVA), which tend to form hydrogen bonds with amino groups and hydroxyl groups on the chitosan chain, promoting crosslinking between chains [74]. PEG and its derivatives, such as PEG ester, PEG sulfonate, PEG acid, PEG aldehyde, PEG acrylate, and PEG iodide, are able to be grafted onto the chitosan backbone to obtain various CS-g-PEG copolymers with improved water solubility and gelation ability [75]. For instance, Bhattarai et al. prepared CS-g-methoxy polyethylene glycol. When the concentration of PEG is between 45% and 55%, chitosan grafting forms a thermally reversible hydrogel. However, excessive PEG grafting (>55 wt%) inhibits the hydrophobic interaction between chitosan chains, resulting in a non-gel solution at 37 °C [76]. Thermally responsive hydrogels are also able to be formed by complexing with PEG and CS or its derivatives.

The CS/PVA complex hydrogel is similar in structure and mechanism to the CS/PEG complex hydrogel. Due to the high hydrophilicity of PVA, it facilitates the improvement of water solubility of CS [77]. As a result, CS is responsible for hydrophobic interactions at high temperatures, reducing hydrogen bonding and promoting gel formation. While PVA is related to hydrogen-bonding interactions at low temperatures, as the PVA content is increased, the longer the gelation time, the higher the LCST, the more tightly entangled the CS chains are with the PVA chains. Therefore, the porous structure of the hydrogel becomes more compact and the pore size is smaller [78]. To enhance the mechanical properties of the CS/PVA hydrogel, hydroxyapatite (HA), the main inorganic component of bone, was introduced. The strength of the HA-CS/PVA composite gel is significantly higher than that of single CS/PVA gel without affecting the thermosensitive properties. CS chains is able to be weakly connected through the PO_4_^3−^ of HA, leading to faster gelation speed and enhanced gel strength. The CS/PVA hydrogel blended with 0.1 mM HA has the lowest degree of swelling, effectively controlling the release rate of loaded proteins [79].

### 4.4. Chitosan/Amphiphilic Polymer Hydrogel

Poly(*N*-isopropylacrylamide) (PNIPAM) is a thermosensitive polymer that exhibits reversible phase transitions with LCST in the range of 30–35 °C [80]. When the temperature exceeds the LCST, PNIPAM solutions undergo a sharp transition from an expanded random-coil state to a compact hydrophobic globular state [81]. Utilizing such properties, PNIPAM can be grafted onto CS chains to impart thermosensitive phase-transition characteristics to CS, while enhancing the mechanical strength of PNIPAM and accelerating its gelation process. For example, Lu et al. prepared PNIPAM-grafted CS (CS-g-PNIPAM) with a temperature transition around 30 °C. Compared to PNIPAM hydrogels, this copolymer exhibited rapid phase-transition kinetics, excellent phase-transition reproducibility, and significant improvement in mechanical strength [82].

Poloxamers or pluronic are amphiphilic triblock copolymers composed of a central hydrophobic polypropylene oxide (PPO) block flanked by two hydrophilic polyethylene oxide (PEO) blocks (PEO-PPO-PEO) [83]. These polymers are well known for forming hydrogels when the temperature is above their LCST. For example, Tohidi et al. prepared thermosensitive CS–pluronic hydrogels by grafting CS onto the terminal groups of pluronic, resulting in hydrogels with improved transparency, stability, biocompatibility, and mechanical properties. The CS–pluronic solution underwent a rapid sol–gel transition at 25 °C [84]. Moreover, Liu et al. successfully introduced Poroxam 407 into chitosan and prepared a temperature-sensitive hydrogel dressing of Poroxam 407/chitosan loaded with oxygen-producing matrix CaO and dihydromyricetin (DHM), which not only created an ideal oxygen environment for the cells around the wound, but also effectively reduced the aggregation of inflammatory cells and excessive collagen deposition. More critically, it stimulated neovascularization and promoted cell proliferation, thereby greatly accelerating the healing process of diabetic wounds [85].

### 4.5. Chitosan/Alkaline Inorganic Salt Hydrogels

Alkaline inorganic salt solutions are contributing to gradually neutralizing the protons on the chitosan chains, causing the pH of the system to slowly raise. With the increase in temperature, the hydrogen-bond interaction between chains is greater than the electrostatic repulsion, and the hydrophobic effect is enhanced, triggering the sol–gel transition [86]. Therefore, the salt content and its pH-adjusting ability play a key role in the sol–gel transition of CS/alkali-type inorganic salt solutions. The CS/sodium bicarbonate (NaHCO_3_) solution is one such example. The NaHCO_3_ solution was slowly added to the chitosan solution at 4 °C to obtain a homogeneous mixture, which showed a sol–gel transition when heated to 37 °C. Gelation achieved with NaHCO_3_ in a moderate concentration range of 0.08 to 0.12 mol/L. When the NaHCO_3_ concentration is 0.07 mol/L, no gel is formed; and at a concentration of 0.13 mol/L, precipitation is formed instead of a gel [87]. Additionally, the gelling time is shortened with increasing NaHCO_3_ content, which affects the ionization equilibrium. Such a process of CS/phosphate gelation is not achieved by the intervention of the system pH, but only by the pH-adjusting ability of the alkaline inorganic salts. For example, Casettari et al. studied the physicochemical and rheological properties of chitosan in the presence of NaH_2_PO_4_ and Na_3_PO_4_ at pH values ranging from 5 to 7, and found that pH and the salt/CS ratio were the two key factors in the thermal gelation process. In particular, at pH 7.0 and a phosphate/CS ratio of 2, in the presence of both salts, the system exhibited gel formation at 45–50 °C, confirming that a pH value close to 7.0 is crucial for the thermal gelation properties of CS/phosphate systems [88].

## 5. The Application of Chitosan-Based Thermosensitive Hydrogels in the Treatment of Different Types of Wounds

**Figure 9:**
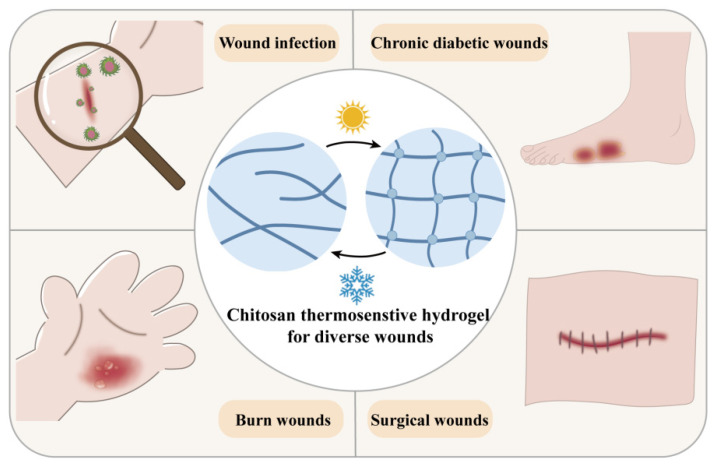
The application of chitosan thermosensitive hydrogels in the treatment of different types of wounds.

The healing of skin wounds is a complex mechanism involving multiple biological processes and signaling molecules [89,90]. To promote wound healing, selecting appropriate wound dressings is crucial. Chitosan-based thermosensitive hydrogels have become an ideal choice for treating irregular wound sites due to their unique temperature sensitivity and injectability. Moreover, these hydrogels serve as carriers for bioactive molecules or drugs, forming complementary dressings with multiple functions [91]. Chitosan-based thermosensitive hydrogels have demonstrated significant application potential in treating various wound types, such as infected wounds, burn wounds, chronic wounds caused by diabetes, and surgical wounds (Figure 9). Table 1 provides a detailed overview of the applications and characteristics of these hydrogels in different wound types.

### 5.1. Infected Wounds

**Figure 10:**
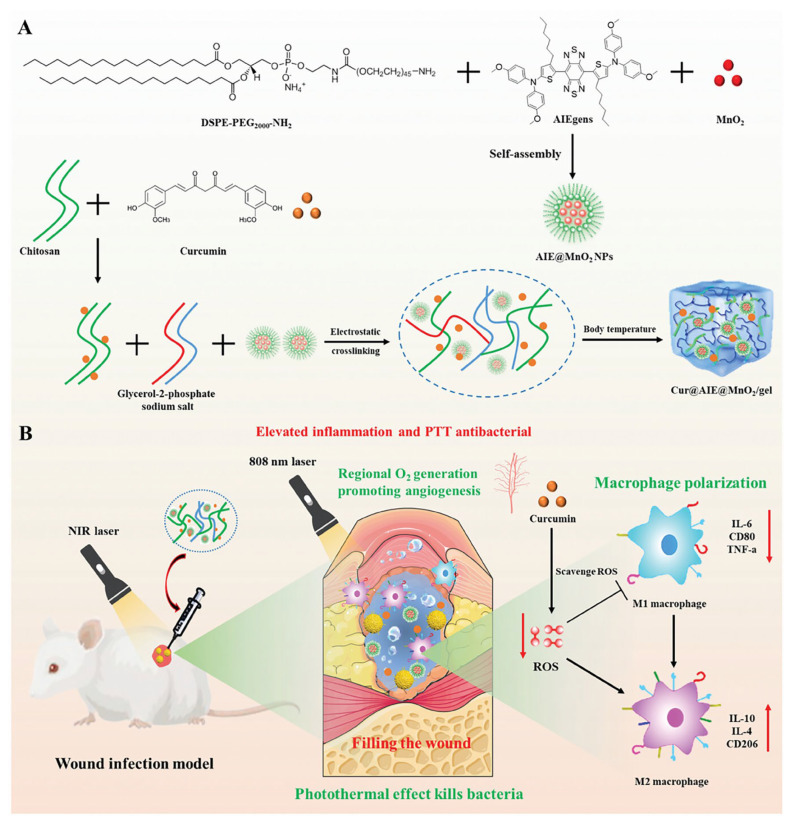
A schematic diagram of Cur@AIE@MnO_2_/gel in skin infection antibacterial therapy. (**A**) The synthesis of Cur@AIE@MnO_2_/gel and (**B**) its application in the treatment of *S. aureus*-infected wound healing [114].

Wound infection is a major obstacle to the healing process and is usually determined by a combination of host immune resistance, wound management methods and invasion of pathogenic microorganisms [108,109]. To combat infections, chitosan-based thermosensitive hydrogels have demonstrated their unique potential. It is possible for these hydrogels to act as drug-delivery systems carrying antimicrobial drugs such as ciprofloxacin and minocycline for localized sustained release, effectively preventing wound infections and promoting normal tissue recovery [110]. Additionally, broad-spectrum antibacterial materials like zinc oxide nanoparticles and lysozyme are considered potential antibacterial components to be incorporated into hydrogels to enhance their antibacterial performance [111]. As an innovative intelligent material, photothermal materials are able to achieve local heating under light excitation, achieving broad-spectrum antibacterial effects [112,113]. For instance, Wu et al. crafted a multifunctional Cur@AIE@MnO₂ hydrogel. They employed chitosan and glycerophosphate as raw materials, added near-infrared responsive AIEgens for photothermal conversion, loaded MnO₂ NPs for catalytic oxygen production, and incorporated anti-inflammatory curcumin. Compared with other groups, in vitro and in vivo experiments showed that Cur@AIE@MnO₂/gel exhibited remarkable efficacy. It was capable of eradicating pathogenic bacteria, mitigating local oxidative stress and inflammation. By achieving sustained oxygen release and stimulating collagen deposition, it promoted angiogenesis. Consequently, it significantly expedited the process of wound repair and tissue regeneration (Figure 10) [114].

### 5.2. Burn Wounds

Wounds in burn patients, regardless of their depth and size, rapidly exude body fluids. Therefore, wound dressings need to have good breathability to maintain an optimal environment for wound healing [115]. Additionally, good hydrophilic properties are crucial for maintaining a moist wound environment, softening necrotic tissue, and promoting skin tissue regeneration [116]. With its high specific surface area and porosity, chitosan-based thermosensitive hydrogel is able to efficiently absorb body fluids exuded from the wound and maintain a high degree of solubility, providing a slightly moist environment for the wound [12]. Such an environment helps to soften necrotic tissue and promotes cell proliferation and migration, which accelerates the wound-healing process. The research by Zhou et al. further confirmed the advantages of chitosan thermosensitive hydrogels. They formulated a chitosan/collagen/β-glycerophosphate (β-GP) thermosensitive hydrogel infused with human umbilical cord mesenchymal stem-cell-conditioned medium (MSC-CM), known as MSC-CM/hydrogel. Their results revealed that this hydrogel not only significantly reduced wound-healing time but also curbed inflammation, enhanced epithelial cell regeneration, fostered the development of high-quality, well-vascularized granulation tissues, and minimized the formation of fibrotic and hyperplastic scar tissues. These findings offer compelling evidence of the efficacy of MSC-CM/hydrogel in promoting wound healing in third-degree burned mice [98].

**Figure 11:**
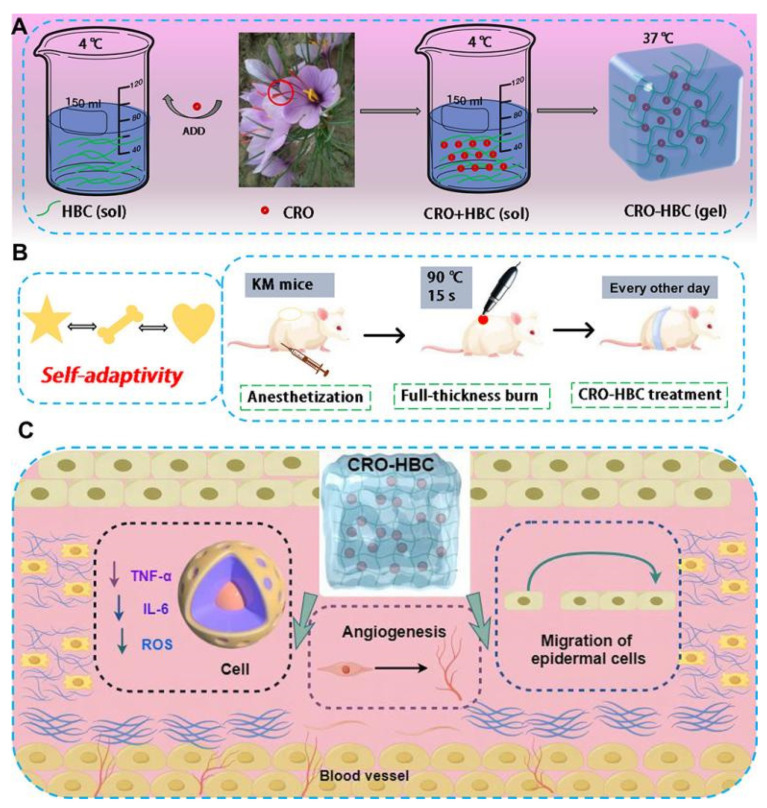
The preparation of temperature-sensitive and self-adaptive CRO-HBC hydrogel for accelerating full-thickness burn healing. (**A**) The fabrication process of CRO-HBC hydrogel and its sol–gel transition processes under temperature increase. (**B**) The self-adaptivity and treatment on a full-thickness burn wound of CRO-HBC hydrogel. (**C**) The multifunctionality of CRO-HBC hydrogel, including providing a physical barrier, enhancing migration, angiogenesis, and anti-inflammatory capabilities [118].

Another significant advantage of chitosan thermosensitive hydrogels is the ability to rapidly gelate at body temperature. This property allows for easy fading by controlling the temperature of the gel solution during dressing changes, which reduces patient pain. Additionally, chitosan-based thermosensitive hydrogel is able to completely cover wounds of various shapes and depths due to its injectable property, providing a more flexible and convenient approach to wound management [13]. Finally, chitosan thermosensitive hydrogel is also biocompatible and is able to be loaded with drugs or bioactive substances that act on the wound through sustained release, further promoting the healing of burn wounds and reducing the frequency of dressing changes [117]. These unique properties make chitosan thermosensitive hydrogel a wide application prospect in the treatment of burn wounds. Lv et al. developed thermosensitive chitosan hydrogels loaded with the active component of saffron-1 with anti-inflammatory activity. The mechanism of action of this hydrogel lies in its ability to effectively reduce the level of reactive oxygen species (ROS) and simultaneously inhibit the overexpression of inflammatory factors such as tumor necrosis factor-α (TNF-α) and interleukin-6 (IL-6), which were particularly suitable for treating severe full-thickness burn wounds (Figure 11) [118].

### 5.3. Diabetic Wounds

Diabetes, a prevalent clinical condition, has an astonishing global reach. As per the International Diabetes Federation (IDF), a staggering 537 million adults worldwide were diagnosed with diabetes by 2021. Wounds in diabetic patients are particularly challenging [119]. These wounds often appear along with a sustained inflammatory response, oxidative stress damage, and impaired angiogenesis, as well as bacterial infection due to hyperglycemic stimuli [120,121]. These issues collectively lead to serious consequences such as skin ulcers, tissue necrosis, and wound infections, which are complex and expensive to treat, posing a pressing medical challenge globally [122]. To address these issues, scientists have been seeking a more ideal hydrogel for diabetic wounds. This kind of hydrogel not only needs to have the basic properties of traditional hydrogel dressings, such as providing a moist healing environment and assisting in self-soluble debridement, but also needs to have multiple functions such as anti-inflammatory, antimicrobial, and antioxidant functions, promoting angiogenesis and lowering blood sugar.

**Figure 12:**
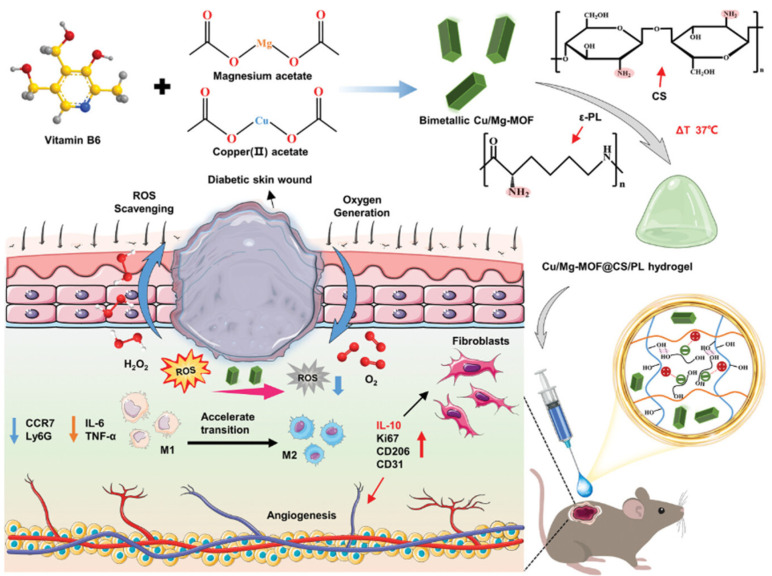
Preparation of Cu/Mg-MOF@chitosan/ε-polylysine hydrogel for diabetic wound repair [126].

During the healing process of chronic wounds, repeated tissue injury elicits an excessive release of cytokines, continually provoking and attracting immune cells to the injury location. This often leads to an overactive inflammatory response, which further hinders the progress of wound healing [123]. A recent study revealed that, in the experimental group, the expression of pro-inflammatory M1-type macrophages was notably lower compared to the control group. Conversely, the expression of M2-type macrophages, which possess anti-inflammatory and pro-repair properties, was significantly higher. This discovery offers novel insights into the treatment of diabetic wounds. Additionally, the researchers developed an innovative thermosensitive hydrogel material composed of chitosan–pluronic F127 (PF127) and loaded with rat adipose-derived mesenchymal stem cells (ADSCs). It was found that the hydrogel significantly promoted the formation of new capillaries, thereby accelerating wound healing through experimental studies on the skin wounds of diabetic rats [124]. Moreover, high concentrations of reactive oxygen species (ROS) are also a significant factor contributing to the difficulty in healing diabetic chronic wounds [125]. To reduce ROS concentrations in wounds, Cai et al. developed a thermosensitive Cu/Mg-MOF@chitosan/ε-polylysine hydrogel, which was able to effectively eliminate ROS and improve the inflammatory microenvironment of wounds, which resulted in promoting the healing of diabetic wounds (Figure 12) [126].

### 5.4. Surgical Wounds

**Figure 13:**
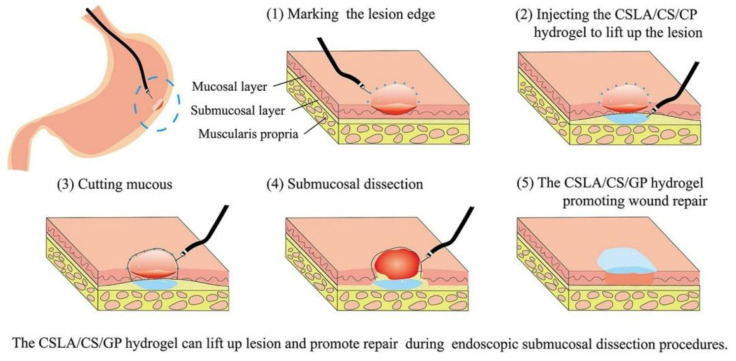
The CSLA/CS/GP hydrogel can lift lesion and promote repair during endoscopic submucosal dissection procedures [128].

With traditional methods of surgical wound closure, such as the use of sutures or staples, these methods may tend to result in poor tissue integration, additional trauma, and the risk of content leakage due to their invasive qualities [127]. To overcome these shortcomings, researchers have developed a novel wound-closure technique based on a thermosensitive hydrogel, which has revolutionized surgical wound healing by virtue of its excellent biocompatibility, tissue adhesion, and temperature sensitivity. These thermosensitive hydrogels not only avoid secondary damage to surgical incisions and reduce the number of dressing changes for patients, but also promote rapid wound healing. In recent years, with the deepening of research, an injectable wound dressing incorporating the concept of bionic design has emerged, which combines excellent mechanical properties, temperature adhesion, as well as self-healing ability. For example, Ni et al. developed lactic acid-modified chitosan/chitosan/β-glycerophosphate (CSLA/CS/GP) thermosensitive hydrogels. Studies have shown that the introduction of lactic acid-modified chitosan (CSLA) significantly enhances the low-temperature fluidity of the precursor solution, enabling it to be manipulated through an endoscopic injection needle. Meanwhile, CSLA also enhances the mechanical strength and bioadhesion of hydrogels, and can be stably preserved for several days in acidic environments, effectively protecting cells. Therefore, CSLA/CS/GP thermosensitive hydrogels were used for wound repair in endoscopic mucosal dissection (ESD) (Figure 13) [128]. In summary, this novel wound-closure technology based on thermosensitive hydrogels not only overcomes many of the shortcomings of traditional methods, but also provides a safer, more efficient, and convenient option for the healing of surgical wounds.

## 6. Conclusions

Chitosan-based thermosensitive hydrogels, as an intelligent polymer material, have demonstrated significant potential in the field of wound repair due to their unique external temperature responsiveness and excellent biocompatibility [129]. This hydrogel exists in an injectable liquid state at room temperature and transforms into a solid state at body temperature. This characteristic not only greatly facilitates drug delivery but also effectively prolongs the duration of drug action at the wound site, providing an ideal environment for wound healing. The application of chitosan-based thermosensitive hydrogel in wound repair has broad prospects, but it still faces a series of challenges [130]. Firstly, the complexity of the preparation process is a major challenge for chitosan thermosensitive hydrogels. Large-scale, high-efficiency, and low-cost preparation processes are crucial for promoting the widespread application of this material. Future research directions should focus on optimizing the preparation process, reducing complexity in the production process and improving production efficiency, while maintaining the excellent performance of the hydrogel. For industrial feasibility, scalable methods like continuous-flow reactors are needed, along with simpler synthesis routes. The standardization of production, quality control, and evaluation protocols is crucial for approval and trust. Cost-efficiency requires exploring alternative chitosan sources or optimizing extraction, streamlining manufacturing, adopting automation, and conducting a cost–benefit analysis considering long-term wound care savings.

Secondly, further enhancing the performance of chitosan-based thermosensitive hydrogels to meet the needs of different wound-repair applications is another challenge. This requires researchers to optimize the performance of hydrogels by adjusting the raw material ratios and introducing novel functional groups to make them more adaptable to various complex wound environments. Future research could explore a wider variety of raw materials and functional groups to develop hydrogels with greater adaptability and more efficient repair capabilities. In addition, safety evaluation for clinical applications is an important challenge for chitosan-based thermosensitive hydrogels. To ensure the safety and efficacy of chitosan-based thermosensitive hydrogels in humans, rigorous in vitro and in vivo experiments and clinical trials are required. Future studies should evaluate the safety and efficacy of chitosan-based thermosensitive hydrogels by conducting more in vitro and in vivo experiments as well as clinical trials in order to provide strong support for clinical applications [131,132].

In summary, in the face of the challenges faced by chitosan-based temperature-sensitive hydrogel in the field of wound repair, it is necessary to explore and innovate from various aspects, such as the preparation process, performance enhancement, and a safety evaluation of clinical application, in order to promote the further development of this material and provide more diversified and precise treatment solutions in the field of wound repair.

## Figures and Tables

**Table 1:** A summary of the application of chitosan-based thermosensitive hydrogel dressings for different wound types.

Application	Name of Dressing	Composition	Tsol-Gel	Gelation Times	Mechanical Strength	Healing Efficiency	Ref.
Infected wound	DG-loaded HP hydrogels	Dipotassium glycyrrhizinate (DG), hydroxypropyl chitosan/*N*-isopropylacrylamide	18.5–23.7 °C	/	Tensile stress: 0.021 MPa	Mouse full-thickness skin defect model: 99.5% average would healing rate at day 14	[92]
	Polydopamine-loaded hydrogels	Polydopamine, chitosan/β-glycerophosphate	37 °C	/	/	Mouse infection wound model: almost complete healing at day 12	[93]
	Dihydromyricetin-loaded hydrogels	Dihydromyricetin, poloxamer/chitosan/hyaluronic acid/	37 °C	0.5 ± 0.2 min	/	Mouse infection wound model: almost complete healing at day 15	[94]
	Bioactive glass-loaded hydrogels	Bioactive glass, quaternized chitosan/PLEL	32.6 °C	/	Adhesion strength: 16.98 ± 0.84 KPa	Practical laceration model: the wound closure reached nearly 99.40% at day 10	[95]
Burn wounds	FA-loaded hydrogels	Ferulic acid (FA), chitosan/ gelatin/glycerol phosphate	37 °C	/	/	Rabbits model of corneal alkali burn: mild corneal hyperplasia at 24 h	[96]
	Nanocurcumin-loaded hydrogels	Nanocurcumin(nCur), chitosan/g-pluronic	35 °C	/	/	Second-degree burn model: complete healing at day 14	[97]
	MSC-conditioned medium-loaded hydrogels	MSC-conditioned medium (MSC-CM), chitosan/collagen/β-glycerophosphate	37 °C	10 min	/	Third-degree burn model: complete healing at day 14	[98]
	Mesoporous carbon nanospheres (MCNs), NO, Sodium nitroprusside (SNP)-loaded hydrogels	MCNs, NO, SNP/chitosan β- glycerophosphate	37 °C	/	/	Rats deep second-degree scald infected model: almost complete healing at day 15	[99]
Diabetic wounds	Insulin and celecoxib-loaded hydrogels	Insulin (INS), celecoxib, polyvinyl alcohol/chitosan/gelatin/phenylboric acid	37 °C	Within 3 s	Adhesive strength 39.36 ± 6.58 kPa	Diabetic rat wound model: the wound-healing rate is 96.68 ± 2.04% on day 14	[100]
	Zinc-mineralized-loaded hydrogels	Zinc-mineralized (ZnDBs), Hydroxybutyl chitosan (HBC)	22.2 °C	/	222.51 ± 19.98 Pa	Diabetic rat wound model: the wound-healing rate is 95.33 ± 0.12% on day 14	[101]
	Nicotinamide mononucleotide-loaded hydrogels	Nicotinamide mononucleotide (NMN), Poluronic F127/Pluronic F68/chitosan	37 °C	80 s	G′: 10 KPa G″: 1 KPa	Diabetic rat wound model: complete healing on the 14th day	[102]
	Chlorogenic acid and deferoxamine-loaded hydrogels	Chlorogenic acid (CGA), deferoxamine (DFO)/chitosan/oxidized hyaluronic acid	RT	/	/	Diabetic rat wound model: the wound-healing rate is 96.5 ± 1.5% on day 14	[103]
Surgical wounds	Black phosphate nanosheets and copper nanoparticles-loaded hydrogels	Black phosphate nanosheets (BPNSs), copper nanoparticles (CuNPs)/chitosan	37 °C	/	/	Mouse infection wound model: almost complete healing at day 10	[104]
	Lactobionic acid-modified chitosan-loaded hydrogel	Lactobionic acid-modified chitosan/chitosan β-glycerophosphate	37 °C	Within 5 min	/	/	[105]
	Galactose modified xyloglucan-loaded hydrogels	Galactose modified xyloglucan (mXG)/hydroxybutyl chitosan	31 °C	20~60 s	/	Rats full-thickness skin defect model: the wound-healing rate is close to 95% on day 14	[106]
	Tannic acid capped gold nanoparticles-loaded hydrogel	Tannic acid capped gold nanoparticles, carboxymethyl chitosan, oxidized fucoidan	37 °C	4.4~5.3 min	Adhesion data: 6.2~9.2 KPa	Mouse infection wound model: the wound-healing rate is 95.8% on day 14	[107]

## Data Availability

No new data were created or analyzed in this study.
